# The Impact of Compounds Released from Damaged Salad Leaves on the Growth and Virulence of *Listeria monocytogenes*

**DOI:** 10.3390/microorganisms13020252

**Published:** 2025-01-24

**Authors:** Asma Alsharif, Lama Aldawsari, Giannis Koukkidis, Primrose Freestone

**Affiliations:** Department of Respiratory Sciences, University of Leicester, University Road, Leicester LE1 7RH, UK; aa1106@leicester.ac.uk (A.A.); lsa2@leicester.ac.uk (L.A.); gianniskoukkidis96@gmail.com (G.K.)

**Keywords:** green salad leaves, *Listeria*, growth, virulence

## Abstract

Background: Fresh produce such as leafy green salads have recently become recognized as a potential source of food-borne infection by enteric pathogens This study investigated whether compounds released from damaged salad leaves were recognized by *Listeria monocytogenes* strain EGD and if they impacted its growth and virulence. Methods: The effects of extracts of salad leaves or salad bag fluids were tested on the growth, biofilm formation, and colonization of salad leaves and host cell virulence. Results: The presence of salad extract at a concentration of less than 0.5% *v*/*v* and salad bag fluids at a concentration of 10% *v*/*v* enhanced the growth in water and serum-based medium by more than 10,000 times over un-supplemented control cultures. Light and scanning electron microscopy, as well as eukaryotic Caco-2 and *Galleria mellonella* models of infection, showed that leafy green extracts from rocket, lettuce, spinach, and their salad bag fluids significantly increased the ability of *Listeria* to establish biofilms and infect host cells. Conclusions: This investigation showed that salad leaf extracts can markedly enhance bacterial virulence, which has implications for bagged salad leaf consumer safety if the leaves become contaminated with pathogenic bacteria such as *Listeria*.

## 1. Introduction

The consumption of fruits and vegetables is associated with healthy lifestyle choices and a reduced risk of developing acute and chronic illnesses such as diabetes mellitus, hypertension, coronary heart disease, stroke, cancer, inflammatory bowel disease, rheumatoid arthritis, asthma, and osteoporosis, as well as potentially aiding in the prevention of obesity [1]. However, fresh produce such as green leafy salads have also become recognized as a potential source of food-borne infection [2,3]. How pathogenic bacteria become associated with fresh produce such as salad leaves is still not fully clear, but leaves might be contaminated before or after harvest from factors such as soil, dust, irrigation, post-harvest human-associated contamination, and washing with contaminated water, as well as the mode of transport, processing (immersion in water and cutting), packing (improper packaging, packing equipment), distribution, and storage temperatures [4]. Harris et al. (2003) [5] showed that free moisture on leaves of growing crops resulting from condensation, rain, or irrigation water may help microbial communities to survive and grow on fresh produce in an otherwise dry and hostile environment. Fresh produce such as green salad leaves can become contaminated with bacteria from soil, contact with feces from birds and grazing animals, or contact with unwashed human hands during processing [6].

One of the most problematic bacterial species associated with food-borne infections is *Listeria monocytogenes*, a cold-adapted pathogen that affects both humans and animals and that can cause potentially serious disease in the immunocompromised or clinically vulnerable [7]. Soil is also the natural environment for *Listeria*, and this species can be spread onto and into the interior of edible plants via uptake by roots or leaf water splashes [8]. *Listeria* is a particularly important pathogen associated with fruit and vegetable infections, largely because such produce is consumed raw, allowing for the more likely development of disease [8]. Leff and Fierer (2013) [9] looked at bacterial communities present on the surface of fresh fruit and vegetables and found that among the families of bacteria associated with the surfaces of different types of fresh produce, *Listeria* was amongst the most frequent microbes that colonized vegetables and green salad, especially ‘ready-to-eat’ mixed-leaf salad. In the USA, outbreaks of listeriosis have been linked to ready-to-eat meat and dairy produce, but recently, *Listeria* infections have been linked to fresh produce such as celery, stone fruit, cantaloupe, and apple [8]. A survey by Szabo et al. (2000) [10] found that *Listeria* could be detected in cut lettuce packages, and a later study by Self et al (2019) [9] showed that that there was *Listeria* present in prepared lettuce and ready-to-eat lettuce in Caesar salad meals.

The widespread prevalence of *Listeria* in the food production environment, its capacity to thrive at low temperatures, and its ability to cause potentially serious disease make this pathogen one of significant concern with respect to the safety of refrigerated and ready-to-eat salad leaf products [9]. In June 2019, within hospitals across the UK, hundreds fell ill, and seven patients died from *Listeria monocytogenes* infection originating from contaminated chicken and salad leaf sandwiches [11]. The *Listeria* outbreak strain was identified as a genetic lineage 1/2a isolate [11,12,13], and the listeriosis infections that occurred were found to be particularly severe [11]. Previously, it was found that fluids released from lettuce and other salad leaves increased the growth and virulence of the enteric pathogen *Salmonella enterica* [3]. As salad leaves were also present in the sandwiches of the 2019 *Listeria* outbreak, the question that arose was whether the salad leaf content may have been contributory to the virulence of the *Listeria*? Using a similar 1/2a strain background (*L.monocytogenes* EGD) [13], the aim of our study was to investigate if salad leaf fluids released after their cutting were also recognized by *Listeria* and if they influenced its growth and virulence.

## 2. Materials and Methods

### 2.1. Bacterial Strains, Culture Media, and Growth Profiling

The bacterial species used in this study was *Listeria monocytogenes* strain EGD serovar 1/2a, a genome-sequenced strain widely used in *Listeria*-based food-related and virulence studies [14,15]. Strain EGD is also of the same genetic lineage as the 2019 UK hospital outbreak isolate [12,13]. A salad microbiota species that we isolated in the current study (*Glutamicibacter arilaitensis*) was also used in some analyses. *L. monocytogenes* EGD and *Glutamicibacter arilaitensis* were routinely cultured in liquid Luria broth medium and Luria agar. For testing growth and virulence responsiveness to the salad juice extract, either a sterile water medium (to mimic the environment within a salad bag) or a host-like serum–SAPI medium [16] were used. Either absorbance at 600 nm or the Miles and Misra method were used to calculate bacterial growth. To calculate *L. monocytogenes* EGD and *Glutamicibacter arilaitensis* colony-forming units (CFUs), a 96-well microliter plate was used; an aliquot (20 μL) of each bacterial culture dilution was mixed with 180 μL of phosphate-buffered saline (PBS) and serially diluted in 10-fold steps. Each dilution was then plated out in 10 μL spots onto Luria agar, followed by incubation overnight at 37 °C in a static incubator.

To determine if salad extract affects *L. monocytogenes* EGD’s uptake of transferrin iron, which is responsible for the bacteriostatic properties of blood and serum, serum–SAPI medium containing ^55^Fe-labelled transferrin (1.5 × 10^5^ cpm/mL, or 1.5 mg ^55^Fe-transferrin/L) was used [16,17]. This medium was then supplemented with 2% sterile salad extracts or an equivalent volume of water (control). *L. monocytogenes* EGD was cultured overnight in Luria broth, its optical density at 600 nm was recorded (Absorbance Units), it was centrifuged at 10,000 rpm for 10 min, then washed in PBS, added at 1 × 10^8^ CFU/mL, and incubated at 37 °C in a static incubator for 18 h. After incubation, the optical density of the cultures at 600 nm was recorded, and the bacteria were harvested by centrifugation at 10,000 rpm for 10 min, washed in PBS, and assayed for ^55^Fe incorporation using scintillation counting, as described previously [17]. The ^55^Fe uptake values shown were normalized for any differences in growth levels between the test and control cultures.

### 2.2. Salad Leaf Extract Preparation

Extracts from iceberg lettuce, spinach, rocket, and mixed salad leaves (lamb’s lettuce, spinach, radicchio, and frisee mixtures) were used in this study. All salad bags were pre-washed and ready-to-eat, and all were processed at least 2 days before their expiration date. To prepare extracts of lettuce or salad leaves, 30 g of leaves was ground for 30 s using a pestle and mortar (to mimic chewing the leaves) [3]. The ground leaf extract was then centrifuged in a sterile 50 mL centrifuge tube for 30 min at 4200 rpm to pellet large particles of plant material. The salad extract was then centrifuged for 10 min at 13,000 rpm to remove smaller pieces of leaf debris to allow for filter sterilization of the resultant liquid. The extracts were sterile-filtered using a 0.2-micron syringe filter and stored at −80 °C. In addition to the salad extract, the bag fluids from spinach, rocket, and mixed salad leaves bags were also utilized. This was carried out using a syringe to collect the excess liquid that typically accumulates at the bottom corners of salad plastic bags [3]. The collected fluids were sterilized through filtration using a 0.2-micron syringe filter and stored at −80 °C.

### 2.3. L. monocytogenes EGD Biofilm Quantification

Biofilm formation involves the binding of microbial cells to surfaces such as living tissues or solid surfaces [18]. In this study, serum–SAPI and nano-pure water were used to examine *L. monocytogenes* EGD biofilm formation on solid surfaces, which was quantified using a crystal violet binding assay. In these experiments, the salad extract was added at concentrations ranging from 0.1% to 5%, and the control was an equivalent volume of sterile water. *L. monocytogenes* EGD from an overnight inoculum grown in LB was diluted in 10-fold steps of 100 μL volumes in triplicate in 96-well plates. The salad-extract-treated test and control cultures were cultured statically for 18 h in a 5% CO_2_ incubator at 37 °C; incubations were also carried out in a refrigerator set at 4 °C for up to 5 days. After incubation, non-attached bacteria and their culture supernatants were removed, and the wells were washed 3 times with 150 μL PBS to remove loosely attached bacteria. After drying at room temperature, 150 μL of crystal violet (0.2% *v*/*v*) was added per well, and the plate left at room temperature for 15 min. Then, the dye was removed, and the wells washed 3 times with 150 μL PBS. The plates were inverted and tapped to remove residual liquid and then left upside-down overnight at room temperature until dry. The next day, a mixture of 80% ethanol and 20% acetone was added to the wells; to elute the crystal violet stain, the plates were placed on a rotating platform for 1 min. Measurement of the surface attachment of the bacteria was carried out at 595 nm using the Microplate Manager 5.2.1 program, which measured the bound crystal violet. As a negative control, crystal violet binding to plate wells containing only water was used to correct the readings for bacterial attachment. Biofilm formation in terms of cell–cell attachment was also analyzed microscopically using a light microscope set at ×40 magnification and recorded using a digital camera [18].

For the analysis of *L. monocytogenes* EGD biofilm formation on salad bag plastic (composed of either polyethylene terephthalate or polypropylene) and salad leaves, scanning electron microscopy (SEM) was carried out. The bag surface was sterilized with 70% (*v*/*v*) ethanol and then cut using sterile scissors into approximately 20 mm by 5 mm sections. For the leaf colonization studies, salad leaves were cut using sterilized scissors into approximately 20 mm by 5 mm sections. These were then placed into sterile water containing no additions (as a control) or 2% salad extract. An overnight *L. monocytogenes* EGD culture grown in Luria broth was inoculated into the water at 10^3^ CFU/mL, and the culture was incubated for up to 24 h at room temperature to represent the likely time duration for salad leaf processing post-harvest [2]. For SEM visualization of the *Listeria* colonization of the salad leaves, respectively, *L. monocytogenes* EGD-inoculated leaf sections were washed three times in PBS and fixed in 2.5% glutaraldehyde in 10 mM Tris–HCl at pH 7.0. After fixation, the leaf sections were washed and dehydrated in a graded series of ethanol to 100%, followed by infiltration with hexamethyldisilane before air-drying. The dry leaf sections were mounted onto aluminum stubs and cool-sputter-coated with gold, and then images were obtained by SEM using a Hitachi S3000H scanning electron microscope, Hitachi, Slough, UK, as described previously [17].

### 2.4. L. monocytogenes EGD Virulence Studies

To investigate the effects of salad extract on *L. monocytogenes* EGD virulence, a *Galleria mellonella* infection model was used [16]. Waxworm larvae were obtained from Live Food UK, Ltd, Axbridge, Somerset, UK. Larvae were starved as recommended for 24 h before being used in the infection studies [19]. A U-100 μL 29G insulin syringe needle from BD Micro-Fine was utilized to inject the worms with *L. monocytogenes* EGD. The *L. monocytogenes* EGD samples were prepared by growing overnight in Luria broth supplemented with 2% (*v*/*v*) salad extract or an equivalent volume of water. After incubation, their optical density at 600 nm was recorded, and then the cells were harvested by centrifugation at 13,000 rpm for 10 min followed by washing once with PBS. After normalizing the optical densities of the cultures, the salad-extract-treated and control *L. monocytogenes* EGD samples were serially diluted in PBS to the required CFU level. Preliminary dose–response trials using a range of inocula suggested that a 10^3^ CFU inoculum gave the clearest distinction between the test and control virulence of the *Listeria* towards the larvae. The *Galleria* larvae were injected intra-hemocoelically with 10 µL of liquid containing 10^3^ CFU of *L. monocytogenes* EGD through the last pro-leg [19]. Controls used were 2% *v*/*v* sterile water or 2% *v*/*v* salad extract only. A 10 µL injection of sterile PBS only was also used as control to account for any potential physical trauma that might be caused by the injection After injection, the larvae were incubated at 37 °C. Each infection experiment had a minimum of 10 worms, and all experiments were performed in triplicate and repeated on at least 2 separate occasions. The worms’ mortality was assessed by tracking the Galleria’s external color for melanization and decreases in motility over time [19].

The impact of the salad extract on *L. monocytogenes* EGD virulence was also investigated using a human Caco-2 epithelial cell line [20]. To study the adhesion to and invasion of the Caco-2 cells by *Listeria*, after washing the bacterial cultures with PBS, an aliquot of *L. monocytogenes* EGD (~5 × 10^6^ CFU) was added to a triplicate set of Caco-2 cells to give a multiplicity of infection of 100:1. For controls, the Caco-2 cells were supplemented with 2% salad extract only or 2% sterile water. The infection assays were incubated in a humidified 5% CO_2_ incubator for 3 h at 37 °C, and after incubation, non-adherent cells were detached by washing the Caco-2 cell cultures 3 times with warm sterile PBS. To enumerate the total number of adhered bacteria, the Caco-2 cells were lysed with 0.5 mL of 1% Triton X-100 mixed with sterile PBS. To calculate the *L. monocytogenes* EGD cells invading the Caco-2 cell line, and from this, the adhered cell fraction, after 3 h of incubation, the *L. monocytogenes* EGD was removed and replaced with pre-warmed PBS containing 300 µg/mL of gentamycin, which killed the adhered bacteria. The gentamycin-treated Caco-2 cells were incubated for 1 h at 37 °C in a 5% CO_2_ incubator and then washed 3 times with PBS to remove the antibiotic. The Caco-2 cells were then lysed with Triton X-100 and lysates serially diluted with PBS and plated onto Luria agar plates and incubated overnight at 37 °C, from which the *L. monocytogenes* EGD attachment and invasion CFU counts were calculated.

### 2.5. Chlorine Inactivation by Salad Leaf Extracts

Chlorine is a widely used salad leaf sanitizer [2,21], and for the analysis of the effect of the salad leaf extracts on chlorine activity, a chlorine bleach with an initial concentration of 5% sodium hypochlorite and 50,000 parts per million (ppm) free chlorine was used. To reproduce the current levels of bleach solutions used worldwide in the sanitizing of fruit and vegetables [2,21], the chlorine solution was diluted to 100 ppm using sterile water. To adjust the chlorine pH to neutrality, HCl was added, as this is the optimal pH for chlorine bleaches [21,22]. A *L. monocytogenes* EGD culture was grown overnight at 37 °C in Luria broth and diluted using sterile water to 10^6^ CFU/mL, and triplicate 1 mL aliquots were added to 24-well plates. The culture wells were supplemented with 2% salad extract or an equivalent volume of sterile water (control). Then, the salad extract and non-supplemented control *L. monocytogenes* EGD cultures were exposed to 100 ppm free chlorine for 10 min at room temperature. After the incubation, the optical density at 595 nm of the bacteria within the cells was measured by using the Microplate Manager 5.2.1 program using an ELISA reader. Before and after the salad extract treatment, the free chlorine levels of the bleach were measured by using chlorine measurement test strips (Quantofix Chlor, Camlab, Cambridge, UK). The pH levels of the chlorine before and after incubation were also measured by applying 2 drops from each culture sample onto Whatman pH strips (Whatman, Maidstone, UK) for 30 s.

### 2.6. The Microbial Content of Bagged Salads

To investigate the spectra of microbiota that are naturally present in salad bags, leaves were analyzed from washed and ready-to-eat mixed-salad and single-leaf salad bags. All salad leaves were used at least 2 days before their expiry date. The microbial presence in the salad leaf bags was visualized by placing the leaves on the surface of Luria agar plates, and these were then incubated at room temperature for up to 72 h [3]. The salad leaves used were spinach and rocket, as these are flattest and attached better to the agar surfaces. Microbial leaf prints of rocket, spinach, and the salad plastic bag interior were also obtained by pressing the sections of the leaf or bag interior on the surface of the Luria agar plate for a minute and then aseptically removing the plastic or leaves. The salad leaf and plastic bag microbial growth profiles were recorded using a digital camera.

We identified some of the more dominant salad leaf microbiota using DNA sequencing. After the colonies were isolated to purity by plate streaking on *Listeria*-selective agar, the salad leaf species were sequenced by Microbes NG (University of Birmingham, UK). Genomic DNA libraries were prepared using a Nextera XT Library Prep Kit (Illumina, San Diego, CA, USA) following the manufacturer’s protocol with the following modifications: the input DNA was increased 2-fold, and the PCR elongation time was increased to 45 s. DNA quantification and library preparation were carried out using a Hamilton Microlab STAR automated liquid handling system (Hamilton Bonaduz AG, Switzerland). Libraries were sequenced on an Illumina NovaSeq 6000 device (Illumina, San Diego, CA, USA) using a 250 bp paired-end protocol. Sequence reads were adapter-trimmed using Trimmomatic version 0.30 with a sliding-window quality cutoff of Q15. De novo assembly was performed using SPAdes version 3.7, and contigs were annotated using Prokka Microbes NG (University of Birmingham, Birmingham, UK).

## 3. Results

### 3.1. Listeria Growth Is Increased by Salad Leaf Fluids

**Figure 1:**
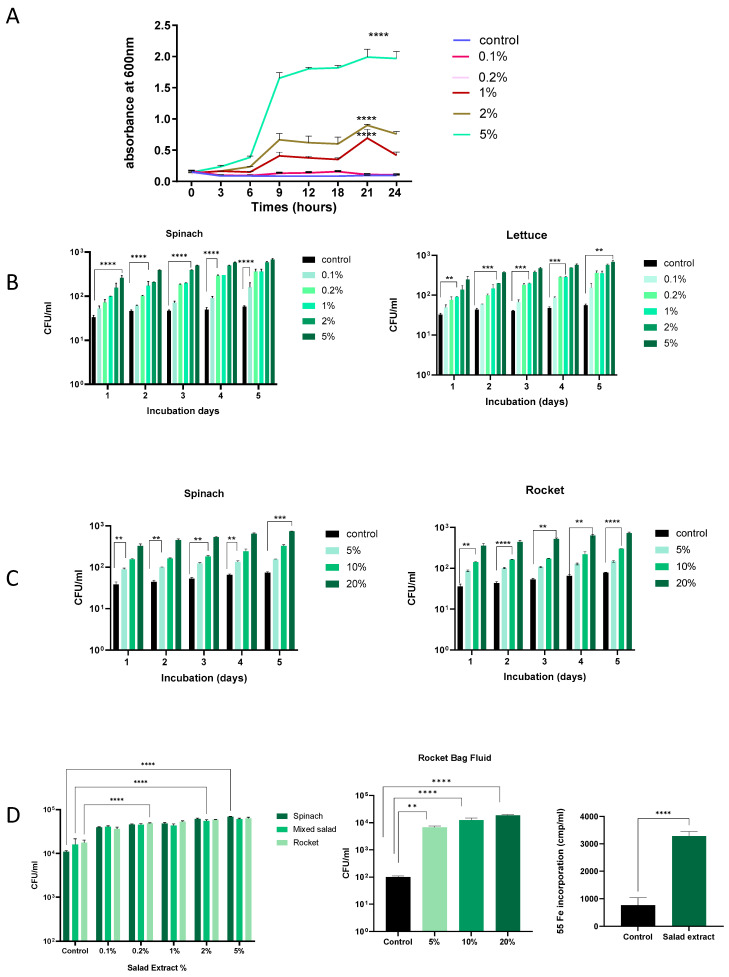
The effect of salad leaf extract and salad bag fluid on *Listeria* growth. Panel (**A**): This time course shows the growth at 37 °C over 24 h of a 10^5^ CFU/mL inoculum of *Listeria* EGD in water with increasing levels of rocket salad extract (0.1% to 5% *v*/*v*) or no additions (control). Error bars show the standard errors of means for three individual experiments. Bacterial numbers were quantified as described in the Materials and Methods section. Values shown are means of triplicate time points (*n* = 3). Key: **** (*p* ≤ 0.0001). Panel (**B**,**C**): A 10^5^ CFU/mL culture of *Listeria* EGD was inoculated into water supplemented with 0.1% to 5% *v*/*v* spinach extract (left panel) or lettuce salad extract (right panel) or, as shown in Panel (**B**), 5%, 10%, and 20% (*v*/*v*) additions of filter-sterilized spinach salad bag fluid (left panel) or rocket salad bag fluid (right panel). Panel (**C**): Controls consisted of equivalent water additions (Control). All cultures were incubated for 5 days at 4 °C, with daily sampling for growth. Error bars show the standard errors of means for three individual experiments (*n* = 3). Values shown are means of triplicate time points. Key: ** (*p* ≤ 0.01), *** (*p* ≤ 0.001), **** (*p* ≤ 0.0001). Panel (**D**): A 10^5^ CFU/mL *Listeria* EGD culture was inoculated into serum–SAPI supplemented with 0.1% to 5% *v*/*v* salad extract (left panel) or 5–20% rocket salad bag fluid (middle panel) or equivalent water additions (control) and incubated for 18 h at 37 °C. Error bars show the standard errors of means for three individual experiments. The right panel shows *Listeria* uptake of Fe from ^55^Fe-labelled transferrin in the absence (control) or presence of 2% rocket extract (salad extract); (*n* = 3). Key: ** (*p* ≤ 0.01), **** (*p* ≤ 0.0001).

Salad leaves can become damaged during processing, and any bacteria present will inevitably come into contact with any leaf fluids released [2,3]. Research has demonstrated that *Listeria* can proliferate more easily in environments where fresh produce is damaged or has been cut [23,24]. We therefore investigated the effect of salad leaf extracts on *L. monocytogenes* EGD growth at different temperatures and in different culture media. The concentration of salad extracts used was from 0.1% to 5% *v*/*v*, and the control was water or no addition of salad extract. We used salad extract made from mixed salad leaves, spinach, lettuce, and rocket. As shown in Figure 1A, *L. monocytogenes* EGD was inoculated at 10^5^ CFU/mL into water with 0.1% to 5% *v*/*v* additions of spinach salad extract or an equivalent volume of water (control) and grown at 37 °C, with the growth recorded by monitoring the optical density at 600 nm over 24 h. It can be seen that concentrations of salad extract above 0.1% significantly increased *L. monocytogenes* EGD growth, indicating that the bacterium was growth responsive to the nutrients within the fluids released from the damaged salad leaves. Nutrient analyses of lettuce, spinach, and rocket leaves show they have a water content of 96, 93, and 93 g/100 g, respectively, and total sugar levels of 1.4, 0.1, and 0.1 g/100 g [25]. Lisiewska et al. (2011) analyzed the protein content of salad leaves such as spinach and found that a wide variety of trace levels of amino acids were present [26]. The range of nutrients available within salad leaves is consistent with the dependency of the growth promotion of *L. monocytogenes* EGD on the concentration of the salad extract in water that we observed.

*Listeria* is well known for its ability to grow at refrigeration temperatures [14], and so we repeated the dose–response analysis in water at 4 °C, testing for increases in *L. monocytogenes* EGD growth every 24 h. Figure 1B shows that over 5 days of refrigeration, the *Listeria* in water for both the spinach and lettuce extract treatment showed a consistent increase in cell number starting from day 1, and by day 5, the cell number increased to over 1 log over the un-supplemented control. Similarly, as shown in Figure 1C, salad leaf bag fluid also stimulated *L. monocytogenes* EGD growth at refrigeration temperatures over time, though due to the higher water content of the bag fluid [2,3], higher doses relative to the leaf extract were needed to stimulate growth. These results suggest that *Listeria* in a salad bag may able to use the nutrients in the fluids within the salad bag released by cut salad leaves to increase their cell number, even at the temperatures present within a domestic refrigerator.

We used serum–SAPI to investigate how *Listeria* might respond to salad extracts and bag fluids when in a host-like medium (Figure 1D). It can be seen that *L. monocytogenes* EGD responded to both the salad extracts and salad bag fluids with significantly increased growth, even though the serum content of serum–SAPI is growth-restrictive due to the presence of iron-sequestering proteins such as transferrin [27]. For *Listeria*, as for many human pathogens, Fe is an essential nutrient [16,27], and so, as shown in Figure 1D, we measured the *L. monocytogenes* EGD uptake of radilabelled iron from ^55^Fe-labeled transferrin in the presence of the salad extract. It can be seen that compared to the un-supplemented control, *L. monocytogenes* EGD used the salad extract to internalize significantly more of the ^55^Fe-labeled transferrin iron. This suggests that if *Listeria* were co-consumed with salad leaves, it would be advantageous to its growth to obtain sequestered host Fe in the iron-restricted environment that is the human gut [27].

### 3.2. Listeria Biofilm Formation and Salad Leaf Colonization Is Enhanced by Salad Extract Exposure

**Figure 2:**
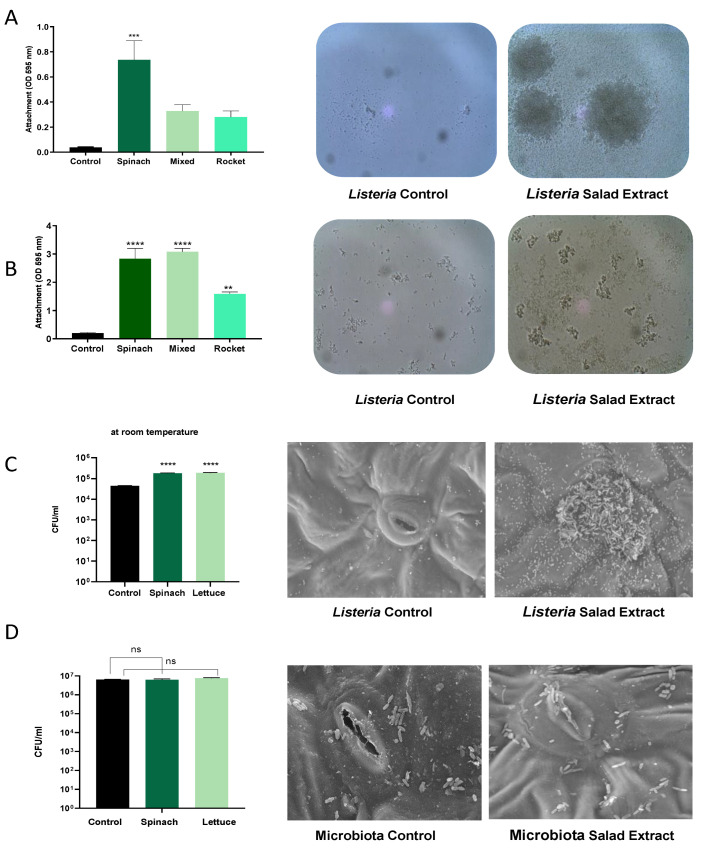
The effect of salad bag extract and bag fluid on *Listeria* biofilm formation and salad leaf colonization. Panel (**A**,**B**): A 10^5^ CFU/mL inoculum of *L. monocytogenes* EGD in water, Panel (**A**), or serum–SAPI, Panel (**B**), was supplemented with no additions (Control) or 2% *v*/*v* salad extracts from spinach, mixed leaves, or rocket; (*n* = 3). The left panels are histograms showing *Listeria* attachment to an abiotic surface after 24 h of incubation in water at 4 °C (Panel (**A**)) or serum–SAPI at 37 °C (Panel (**B**)). Error bars show the standard errors of means for three individual experiments. The middle and right panels show the appearance of *Listeria* cultures in water (Panel (**A**)) or serum–SAPI (Panel (**B**)) when supplemented with equivalent water additions (Control) or 2% *v*/*v* salad extract (Salad Extract) (spinach extract is shown). Bacterial numbers were quantified as described in the Materials and Methods section. Key: ** (*p* ≤ 0.01), *** (*p* ≤ 0.001), **** (*p* ≤ 0.0001). Panel (**C**): A 10^5^ CFU/mL *L. monocytogenes* EGD culture was inoculated into sterile water supplemented with 2% *v*/*v* spinach or 2% *v*/*v* lettuce salad extract (Salad Extract) or 2% of water (Control) containing either spinach or lettuce salad leaf sections and incubated for 45 min at room temperature, as described in the Materials and Methods section. Bacterial attachment to the leaves was counted using *Listeria*-selective agar. Error bars show the standard errors of means for three individual experiments; *n* = 3. Key: **** (*p* ≤ 0.0001). The middle and right panels are scanning electron micrographs showing how the presence of 2% spinach extract significantly increased the *Listeria* colonization of the spinach salad leaves relative to the untreated control; *n* = 4. Panel (**D**) shows similar investigations to those in Panel C but using a 10^6^ CFU/mL culture of *Glutamicibacter arilaitensis.* The histograms and SEM images show that the salad leaf microbiota species was not responsive to salad leaf extract. Key: ns not significant.

As attachment to surfaces is key to the persistence mechanisms of *Listeria* on fresh produce [23], we investigated the impact of the salad extract on the formation of biofilms by *L. monocytogenes* EGD on abiotic and biotic surfaces. Salad bag containers are usually made of plastic, and so we assessed the attachment to a plastic surface using crystal violet staining in the presence of spinach, mixed salad leaves, and rocket salad extracts with water and refrigeration at 4 °C as the culture conditions (Figure 2A) or with serum–SAPI at 37 °C (Figure 2B). The data in Figure 2 show that in both the refrigerated water and serum medium at human body temperature, the salad extracts markedly enhanced the *L. monocytogenes* EGD attachment to the plastic. The spinach leaf extract seemed to stimulate the highest level of attachment in water, and it was equal to the mixed-leaf extract in the serum–SAPI medium. The images of the *Listeria* water and serum–SAPI cultures in Figure 2A,B show that cell–cell association (visible as clumping) was also enhanced by the salad extract compared to the un-supplemented controls. This observation then led us to investigate if biofilm formation on salad leaves was also enhanced when salad extracts were present. Figure 2C shows that the attachment of the *L. monocytogenes* EGD to the salad leaves (the results for spinach are shown) was increased, as was their subsequent colonization of the salad leaf. The scanning electron micrographs show that the *Listeria* tended to form most of its biofilm in the region of the stomata, a finding that has been demonstrated for other salad-leaf-colonizing pathogens [2,3,23,24].

In our salad leaf colonization study, an abundant salad leaf microbiota was always present when the *L. monocytogenes* EGD colonized the salad leaves, but as can be seen in the SEM micrographs shown in Figure 2D, the endogenous salad leaf microbiota isolate *G. arilaitensis* showed no significant increases in attachment to the leaves when the salad extract was present. We also found that unlike *L. monocytogenes* EGD, the salad leaf microbiota species did not show increases in growth when any of the salad extracts were provided (left histogram panel, Figure 2D). This suggests the salad leaf microbiota may have adapted to the fluids released from damaged salad leaves over time, which could promote leaf colonization by more salad-extract-responsive pathogens such as *Listeria*.

### 3.3. Salad Bag Microbiota Studies

**Figure 3:**
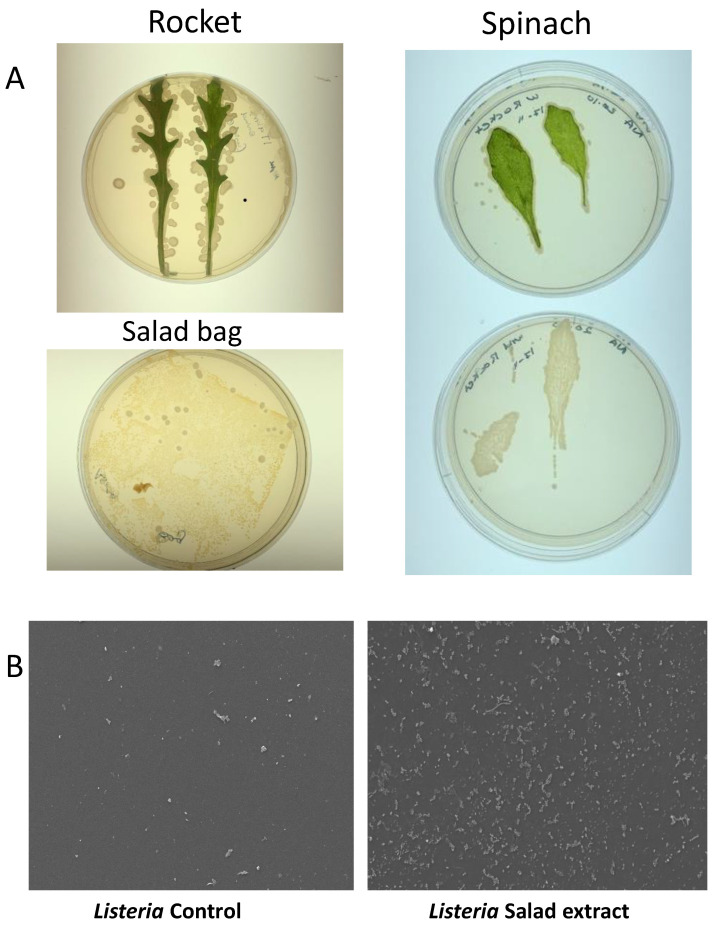
The salad leaf and salad bag microbiota. Panel (**A**): Images showing typical photographs of the microbiota on rocket and spinach salad leaves. The lower left image shows the microbes colonizing the interior surface of the plastic salad bag. Panel (**B**): SEM micrographs revealing that *Listeria* attachment to the salad bag in the absence of salad extract (Control) was highly enhanced when salad extract was present (Salad extract); *n* = 3.

Salad leaves are known to be colonized by a variety of microorganisms [2], and some microbiota profiles and prints of rocket and spinach leaves on Luria agar can be seen in Figure 3A. Although we did not find any *L. monocytogenes* in our search, we did find several colonies that showed as positive on the *Listeria*-selective agar but that were not found to be pathogenic upon DNA sequencing. Table 1 shows the identity of the salad leaf microbiota isolated from the salad bags. *Listeria weihenstephanensis* is a partially anaerobic, non-motile bacillus. Although it shows 79.6% sequence similarity to *L. monocytogenes,* it does not normally cause human disease except in the immunocompromised [28]. *Glutamicibacter arilaitensis* is non-pathogenic to humans and has been found in a variety of artisan French cheeses [29]. The *Marinilactibacillus* isolate was identified as a member of the *Lactobacillaceae* family that produces lactic acid. The presence of *Marinilactibacillus* is often seen in fermented vegetables such as coleslaw [30].

Salad leaves inevitably come into contact with the interior of their salad bag container, and in Figure 3A, the bottom left image shows the microbes cultured from the imprint of the interior of a section of a spinach plastic salad bag. We therefore were curious as to whether the microbiota-colonized salad bag could be a vehicle for *Listeria* attachment. We aseptically sectioned an empty salad bag and incubated *L. monocytogenes* EGD with the bag section in water with or without salad extract for 24 h at 4 °C. The SEM images in Figure 3B show that despite the fact that the microbes were already attached to the salad bag, *L. monocytogenes* EGD was not competitively excluded by them from attaching to the salad bag surface.

The table shows that of the three species of bacteria that were found in the different salad bags, *L. weihenstephanensis* was the most closely related in sequence to *L. monocytogenes*.

### 3.4. Listeria virulence Is Enhanced by Salad Extract Exposure

**Figure 4:**
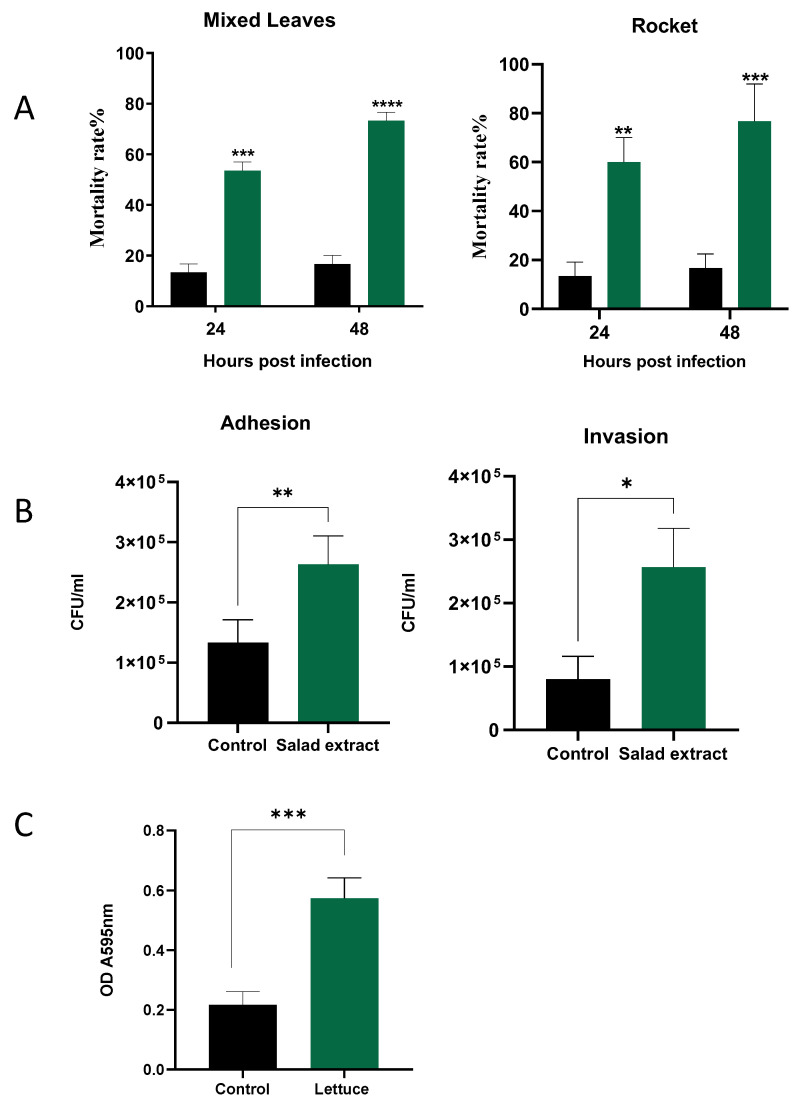
The effect of salad extract on *Listeria* virulence. Panel (**A**): Histograms showing the virulence of control (black bars) and 2% salad extract-treated *L. monocytogenes* EGD (spinach is shown) (green bars) injected into *Galleria* 24 and 48 h after infection (*n* = 3). Data are expressed as percentage survival and are the representative results of at least three independent experiments. Key: ** (*p* ≤ 0.01), *** (*p* ≤ 0.001), **** (*p* ≤ 0.0001). Panel (**B**): The attachment to and invasion of Caco-2 cells by untreated *Listeria* (Control) and spinach salad extract-treated *Listeria* (Salad Extract) after 3 h of incubation at 37 °C. Assays and cell counts were carried out as described in the Materials and Methods section. Key: * *p* < 0.05, ** *p* < 0.01; *n* = 3. Panel (**C**): The 5% salad extract protected *Listeria* from a lethal chlorination treatment. Key: *** *p* ≤ 0.001; *n* = 4. The chlorine inactivation results for the spinach salad extract are shown, but all salad leaf extracts were equally potent in inactivating the chlorine.

The biofilm enhancement of *L. monocytogenes* EGD by the salad extract on the salad leaves suggests that such a treatment might also enhance the attachment to host cells, which is a pre-requirement for *Listeria* infection of a host [15,31]. *Galleria mellonella* was used to examine the impact of salad extract exposure on the virulence of *L. monocytogenes* EGD. The larvae were injected, as described in the Materials and Methods section, with 10 µL of 10^3^ *L. monocytogenes* EGD with salad extract or with sterile water as a control; the inoculated larvae were then incubated at 37 °C for up 48 h. The salad extract used in the infection studies was prepared from mixed leaves and rocket, although spinach and lettuce extract also produced similar results to those shown in Figure 4 (data not shown). Plots of *Galleria* mortality at 24 and 48 h after infection of the larvae are shown in Figure 4A. It can clearly be seen that the treatment with either of the salad extracts (green histograms) increased the *Listeria* virulence compared to the untreated controls (black histograms). Note that the salad extract by itself was not toxic to the *Galleria*.

The biology of humans is different to that of *Galleria* [19], and so we also examined the impact of salad extract exposure on the adhesion and invasion of human gut cells, specifically, the Caco-2 colon cell line [20]. The pathogenic properties of *L. monocytogenes* are dependent on its ability to transverse host cellular barriers and replicate within host cells [15,31]. Figure 4B shows that the presence of the salad extract resulted in a small but reproducible increase in the level of *Listeria* adhesion to and invasion of the Caco-2 cells. *L. monocytogenes* strain EGD is known to be one of the more virulent *Listeria* lineages, and so the limited effect of the salad extract may have been due to this *Listeria* strain’s innate high ability to invade and replicate within host cells [15,31].

Washing leafy salad leaves with chlorinated water is used to reduce pathogenic bacteria levels to 1 to 2 log CFU/g [21,22]. It was noticed in the current study that every salad bag fluid tested was found to be stimulatory to the growth of *L. monocytogenes* EGD, even though the bag fluid should have been inhibitory, as the salad leaves had presumably been previously disinfected with chlorine bleach. As shown in Figure 4C, it was therefore investigated whether the presence of the salad extracts was protective for *Listeria* during chlorination treatment. A *L. monocytogenes* EGD culture was inoculated into water supplemented with 5% salad extract or 5% water as a control and then treated with 100 ppm chlorine, the level used to sanitize fresh produce [21,22]. The results in Figure 4C clearly show that the salad extract helped the *Listeria* survive the lethal level of chlorine. The inactivating effect of the salad extract on the chlorine’s efficacy was also rapid, with chlorine levels dropping from 100 ppm to around 2–3 ppm after only 10 min of incubation. Organic matter can inactivate chlorine bleaches, as can reducing the pH level of the chlorine [2,25]. The bleach was originally pH 7 but decreased to pH 5 after adding the salad extract. Current standards suggest that a pH of 7 is required to adequately chlorine-sanitize fresh produce, so either the organic matter content or the fall in pH may explain *L. monocytogenes* EGD’s chlorine resistance when in the presence of the salad leaf extract. This also explains why the watery fluid within a salad bag was stimulatory rather than inhibitory to the growth of *L. monocytogenes* EGD.

## 4. Discussion

This study investigated what happens when *Listeria* comes into contact with fresh ready-to-eat salad leaves, which are widely used in commercially produced sandwiches and which were present in the 2019 UK *Listeria* hospital outbreak [11,12]. Cutting or chewing salad leaves releases their internal fluid content, and our investigation found that the compounds released from damaged salad leaves can act as potent stimulators of *Listeria’s* growth, its biofilm formation, and importantly, as far as infection by *Listeria* is concerned, its virulence. The *Listeria* EGD strain used in our study is of a similar clonal lineage to the 2019 hospital outbreak *Listeria*, which means that our findings have relevance to how the hospital *Listeria* isolate may have responded to compounds released from the salad leaves in the contaminated sandwiches. We also found that the salad leaf compounds could increase the growth and virulence of another enteric pathogen, *Salmonella enterica* [3], which collectively emphasizes the importance of preventing contamination of fresh salad leaves by pathogenic bacteria.

We found in our study that while salad leaves naturally have a varied microbial population, most of which come from their growth and post-harvest processing environments [2,10,32], the resident salad microbes did not act as a competitive inhibitor of *Listeria* colonization of the salad leaves or bag surface. Importantly, the chlorine bleach that is used to sanitize salad leaves was found to be inactivated by the fluids from the cut leaves, and so it became ineffective in terms of the eradication of any pathogens present. *L. monocytogenes* EGD was actually protected from the chlorine killing by the salad extract inactivating the disinfectant. Listeriosis can be a deadly infection to the immunocompromised and clinically vulnerable, and so it is imperative that *Listeria* is prevented from entering bagged salads. This could be achieved through improved horticultural monitoring for pathogens and more stringent hygiene practices during salad leaf processing.

## Figures and Tables

**Table 1:** Identification of salad bag microbiota.

Family	% Sequence Similarity to *L. monocytogenes*	Species
*Listeriaceae*	79.60	*Listeria weihenstephanensis*
*Micrococcaceae*	13.52	*Glutamicibacter arilaitensis*
*Carnobacteriaceae*	10.32	*Marinilactibacillus* sp.

## Data Availability

The original contributions presented in the study are included in the article, further inquiries can be directed to the corresponding author.
